# Determinants of ventilatory inefficiency in transthyretin cardiac amyloidosis: The role of excessive ventilatory drive

**DOI:** 10.3389/fphys.2022.1002238

**Published:** 2022-10-21

**Authors:** Astrid Monfort, Eugenie Thevenet, Mickael Rejaudry Lacavalerie, Rishika Banydeen, Jocelyn Inamo, Remi Neviere

**Affiliations:** ^1^ Department of Cardiology, CHU Martinique (University Hospital of Martinique), Fort de France, France; ^2^ Cardiovascular Research Team EA7525, Université des Antilles, Fort de France, France; ^3^ Department of Clinical Physiology, CHU Martinique (University Hospital of Martinique), Fort de France, France; ^4^ Department of Clinical Research, CHU Martinique (University Hospital of Martinique), Fort de France, France

**Keywords:** cardiac amyloidosis, cardiopulmonary exercise testing, oxygen kinetics, transthyretin, ventilatory efficiency

## Abstract

**Background and objective:** Along with impaired aerobic capacity, increased slope of the relationship between ventilation (V_E_) and pulmonary CO_2_ output (VCO_2_), i.e., V_E_-VCO_2_ slope is a common finding in patients with cardiac amyloidosis (CA), which suggests ventilatory inefficiency. Little is known about mechanisms leading to ventilatory inefficiency in CA patients. The purpose of this investigation was to examine the factors that underlie the abnormal ventilatory efficiency in transthyretin hereditary CA patients, such as excessive ventilatory drive, inability of pulmonary blood flow to increase adequately during exercise and excessive sympathetic stimulation, which are known mechanisms of V_E_-VCO_2_ slope increase.

**Methods:** In this single-center retrospective observational study, consecutive patients (*n* = 41) with known familial transthyretin amyloidosis *p*.Val142Ile mutation carriers with confirmed cardiac phenotype were included.

**Results:** Compared with CA patients without ventilatory inefficiency (VE-VCO2 slope < 36), patients with ventilatory inefficiency (VE-VCO2 slope ≥ 36) had increased inter-ventricular septum thickness, lower VO_2_ peak along with hyperventilation, and prolonged post-exercise heart rate recovery. By multivariate analysis, only excess of minute-ventilation at anaerobic threshold (*β* = 0.127; *p* = 0.011) remained an independent predictor of ventilatory inefficiency.

**Conclusion:** Our data suggest that high ventilatory stimulation during exercise leading to hyperventilation is the main determinant of ventilatory inefficiency in hereditary transthyretin cardiac amyloidosis patients. This novel finding helps to better understand the mechanism of exercise intolerance in these patients where physiological limitation may be related to both heart dysfunction and abnormal pulmonary response.

## Introduction

Cardiac amyloidosis (CA) is a rare cause of restrictive cardiomyopathy which results from extracellular deposition of misfolded proteins (Wechalekar et al., 2016; Maurer et al., 2019). CA amyloidosis is predominantly considered as a diastolic disease caused by increased myocardial stiffness and restrictive heart wall chamber behavior, along with poor systolic myocardial performance (Wechalekar et al., 2016; Clemmensen et al., 2017; Maurer et al., 2019). Impaired myocardial contractile reserve has also been described using either invasive monitoring or O_2_ kinetics-derived parameters (Clemmensen et al., 2017; Monfort et al., 2020). Poor aerobic capacity evaluated by maximal O_2_ uptake (VO_2_) and ventilatory inefficiency suggested by elevated slope of the relationship between ventilation (V_E_) and pulmonary CO_2_ output (VCO_2_) are commonly observed in exertional patients with CA (Hein et al., 2018; Yunis et al., 2019; Monfort et al., 2020; Bartolini et al., 2021; Bhutani et al., 2021; Dalia et al., 2021; Banydeen et al., 2022a).

In patients with chronic heart failure, V_E_-VCO_2_ slope has been attributed to high physiological dead space ratio (V_D_/V_T_ ratio) during exercise, which may be due to combined effects of pulmonary ventilation-to-perfusion V_A_/Q mismatch and high ventilatory drive (Woods et al., 2010; Weatherald et al., 2018; Phillips et al., 2020). While true pulmonary ventilation-to-perfusion V_A_/Q mismatch is uncommon in patients with mild to moderate chronic heart failure without overt coexisting lung disease, blunted response in cardiac output to exercise will tend to augment V_A_/Q ratio and V_E_-VCO_2_ slope (Woods et al., 2010; Guazzi, 2014). Right ventricular dysfunction and impaired pulmonary hemodynamics can further lead to V_D_/V_T_ ratio increase because regions of ventilated lung remain under perfused during exercise (Lewis et al., 2008; Methvin et al., 2011). Beside underperfusion of ventilated lung alveoli, increased ventilatory drive can also lead to inefficient ventilatory in patients with chronic heart failure. Indeed, patients with chronic heart failure can display rapid breathing and reduced tidal volume V_T_ in response to exercise contributing to hyperventilation and elevated V_D_/V_T_ ratio and V_E_-VCO_2_ slope increase (Weatherald et al., 2018). Likewise, abnormal ventilatory response to exercise in these patients has been attributed to imbalance between sympathetic and parasympathetic stimulation, which can be easily studied by post exercise heart rate recovery (Michael and Graham, 2017; Weatherald et al., 2018).

In patients with CA, little is known about the mechanisms leading to ventilatory inefficiency. The purpose of this investigation was to examine the factors that underlie the abnormal ventilatory efficiency in transthyretin hereditary CA patients, such as excessive ventilatory drive, inability of pulmonary blood flow to increase adequately during exercise and excessive sympathetic stimulation, which are known mechanisms of V_E_-VCO_2_ slope increase. In our study, ventilatory overdrive, blunted early onset of blood flow increase and sympathetic overstimulation during exercise were noninvasively assessed by increased ventilation at anaerobic threshold (excess V_E_@ATVO2), prolonged mean response time (MRT) of VO_2_ increase at the start of exercise, and poor heart rate recovery, respectively.

## Materials and methods

### Patients

In this single-center retrospective observational study, consecutive patients with known familial transthyretin amyloidosis *p*.Val142Ile mutation carriers with confirmed cardiac phenotype were included. The study was performed at the Department of Cardiology, Martinique University Hospital, France from September 2019 to May 2021. All patients were managed in accordance with the amended Declaration of Helsinki (http://www.wma.net/en/30publications/10policies/b3/) and gave their informed consent for the processing of personal data for scientific research purposes. The study was approved by the hospital’s institutional review board (IRB #01022019). Risks and description of the different procedures were explained to the patients, who confirmed their verbal informed consent to enter the study at the time when referred to the cardiovascular department for routine functional evaluation. As a follow-up to our previous work (Monfort et al., 2020), we have included a *de novo* series of CA patients.

### Cardiac amyloidosis diagnosis

All patients declared African ancestries and gene sequencing displayed the transthyretin *p*.Val142Ile mutation. Systemic transthyretin amyloidosis was confirmed in all patients by histological demonstration of amyloid fibrils in salivary duct gland, subcutaneous adipose tissue or endomyocardial biopsies. Cardiac involvement was confirmed by nuclear imaging (General Electric Medical Systems SPECT gamma camera Discovery) showing cardiac uptake grade ≥2 of the Perugini classification of bone tracer technetium-99m-labeled hydroxy methylene diphosphonate [(99mTc)-HMDP]. Cardiac echography revealed the presence of left ventricular hypertrophy and abnormal myocardial texture characterized as a speckled appearance. Abnormal myocardial texture was defined by granular sparkling of the myocardial walls on echocardiography. Due to restricted availability of myocardial imaging techniques, longitudinal strain and resonance imaging were not performed. Cardiac biomarkers included serum high-sensitivity troponin and NT-proBNP. Only patients with NYHA functional class II or higher status were enrolled in the study.

### Echocardiography

Transthoracic echocardiography was with commercially available ultrasound machines (Vivid E9 system from GE Vingmed and IE33 from Philips Norway) using a 2.5 MHz transducer. Two-dimension guided TM tracings, 2D and 3D cardiac loops, Doppler imaging, were generated at optimal time and spatial resolution and synchronized to the electrocardiogram. Cardiac loops were obtained during breath hold. All the images were recorded for off-line analysis. A single observer, blinded to clinical diagnose, performed image analysis. Interventricular septal and posterior wall thickness was measured in 2D-guided M-mode in agreement with the American Society of Echocardiography recommendations. LV end-diastolic and -systolic volumes were measured using Simpson’s biplane method. Left ventricular hypertrophy was defined by wall thickness >12 mm. The mitral inflow velocity pattern was recorded from the apical 4-chamber view with pulsed-wave Doppler sample volume, which was positioned at the tips of mitral leaflets during the diastolic time. Deceleration time, peak velocities of E and A waves, and mitral annulus lateral Ea velocity were averaged over three cardiac cycles. Parameters of right ventricular systolic function included tricuspid annular plane systolic excursion (TAPSE), peak systolic tissue Doppler velocity of the tricuspid annulus pulsed Doppler S wave (RV Sm) and fractional area change (RV-FAC).

### Pulmonary function

Pulmonary function was performed on the request of patients’ cardiologists. Standard forced expiratory spirometry (forced expiratory volume in the first second (FEV1) and forced expiratory vital capacity (FVC) were evaluated (LF8, Ganshorn Medizin Electronic GmbH, Niederlauer, Germany) according to the European and American Thoracic Society guidelines (Graham et al., 2019). Lung volume was considered as normal FEV1/FVC ≥ 0.70 and FVC ≥ 80% of predicted values. Restrictive spirometry pattern was defined as FEV1/FVC ≥ 0.70 and FVC < 80% predicted values.

### Cardiopulmonary exercise testing

All participants performed a cardiopulmonary exercise testing using an electromagnetic upright braked cycle ergometer following the ATS/ACCP recommendations (American Thoracic Society et al., 2013). Unstable cardiovascular diseases, orthopedic impairment that compromises exercise performance, and mental impairment leading to inability to cooperate contraindicated the cardiopulmonary exercise testing. Exercise protocol involved an initial 3 min resting condition, followed by unloaded cycling for 2 min. A progressive increment (10 W/min) until exhaustion at a pedaling frequency of 60–65 rpm was then applied. A 12-lead ECG (Case, GE Healthcare, France) was continuously recorded. Blood pressure was determined every 2-min. Breath-by-breath cardiopulmonary measurements (PowerCube-Ergo, Ganshorn Medizin Electronic GmbH, Niederlauer, Germany) were recorded at rest, unloaded warm up and during incremental exercise testing. Subjects breathed through an oro-nasal mask (Hans Rudolf 7450 SeriesV2™ Mask, CareFusion, France). Oxygen (O_2_), carbon dioxide (CO_2_) sensors, and flow mass sensor were calibrated before each test using precision gas mixture and a 3-L syringe, respectively. VO_2_ kinetics was first assessed during 3 min of unloaded exercise.

The rate of VO_2_ increase during unloaded cycling was expressed as the mean response time (MRT) for a mono-exponential curve fit to the 10 s-by-10 s VO_2_ measurements during the 3 min of unloaded cycling (Chatterjee et al., 2013). MRT is thus the exponential time constant of VO_2_ onset kinetics and approximates the time needed to reach 63% of steady-state VO_2_ (Chatterjee et al., 2013).

Minute ventilation (V_E_), oxygen uptake (VO_2_), carbon dioxide output (VCO_2_) were recorded as concurrent 10-s moving averages. The ventilation anaerobic threshold was determined by the V-slope method. Ventilatory reserve was calculated as ((MVV–peakV_E_)/MVV*100), where MVV is maximal voluntary ventilation calculated as FEV_1_ multiplied by 35. Predicted V_E_ at anaerobic threshold (AT) was calculated as V_E_@AT = 21.8*VO_2_ + 5 (Jones and Campbell, 1982; Fairshter et al., 1987; Péronnet et al., 2007; Péronnet and Aguilaniu, 2014) and excess ventilation at anaerobic threshold (excess V_E_@AT_VO2_) was expressed as percent increase of predicted V_E_. Ventilatory efficiency, as indicated by V_E_ relative to VCO_2_ (V_E_-VCO_2_ slope) rise was calculated off-line as a linear regression function using 10-s averaged values. The non-linear part of the relationship after the respiratory compensation point (where non-linear rise in V_E_ occurred relative to VCO_2_ in the presence of decrease of end-tidal pressure of CO_2_) was excluded for V_E_-VCO_2_ slope calculation (Sun et al., 2002). According to the EACPR/AHA recommendations for the prognostic and diagnostic stratification for patients with heart failure, a value of 36 for V_E_-VCO_2_ slope was used as a cut-off for ventilatory inefficiency (Guazzi et al., 2012). Exercise oscillatory ventilation (EOV) was defined as a persistence of cyclic oscillatory ventilation pattern for at least 60% of exercise at an amplitude ≥15% of the average resting value (Guazzi et al., 2012).

Borg-perceived exertion ratings for both respiratory and leg discomfort were assessed at peak exercise in all subjects. Participants were encouraged to continue exercise cycling until a true symptom-limited exhaustive level was achieved. As recommended by the ATS/ACCP (American Thoracic Society et al., 2013), an effort was considered as maximal if two of the following criteria occurred: predicted maximal work achieved, age-predicted maximal heart rate (HR_max_) achieved, ventilatory O_2_ equivalent V_E_/VO_2_ > 45, and respiratory exchange ratio (RER, i.e. volume of carbon dioxide produced/volume of oxygen consumed) >1.10. Immediately after peak exercise, participants underwent a 3-min cool-down. Heart rate (HR) recovery, measured as beat per minute (bpm), was defined as the difference between the highest observed HR (peak HR) during the graded exercise test and the heart rate measured at 1 min and 3-min of active recovery (Bailey et al., 2018). VO_2_ recovery kinetics was assessed by calculating the time from the end of loaded exercise until the VO_2_ permanently falls below peak VO_2_, i.e., VO_2_ recovery delay (Lauer, 2009).

### Statistical analysis

Normally distributed data are presented as mean ± standard deviation (SD). Categorical data are presented as absolute values with percentages. Between group differences were assessed using *t*-test and Chi-square for normally distributed data and dichotomized data, respectively. Relationships between quantitative variables were assessed by the Spearman’s correlation coefficient. The role of key variables associated with ventilatory efficiency was tested by univariate and multivariate linear regression. Interaction terms were also tested. Variables with a *p*-value < 0.20 after univariate analysis were included in the multivariate model (forward). Goodness-of-fit of the final multivariate model was ascertained by the conditions of normality, homocedasticity and independence between observations, as assessed respectively by the tests of Shapiro-Wilk, White, and Durbin-Watson. Statistical analyses were performed using the Statistical Package for the Social Sciences (SPSS) version 18.0 for Windows (SPSS, Inc., Chicago, IL). A two-sided significance level of 0.05 was chosen for all tests.

## Results

Main demographics of patients with *p*.Val142Ile transthyretin cardiac amyloidosis are summarized in Table 1. Of the 46 eligible patients with cardiac amyloidosis, 5 patients were excluded due to cessation of the exercise protocol without apparent motive. Therefore, the final sample consisted of 41 patients who underwent clinical, biological and functional evaluation. The mean age of the cohort was 73 ± 7 years. All patients were symptomatic ranging from mild shortness of breath and slight limitation during ordinary activity to marked limitation in activity due to dyspnea and/or angina (NYHA II, III, and IV) with high NT-proBNP levels. Most patients received loop diuretics, whereas none had beta-blockers. Transthoracic echocardiography revealed reduced LV ejection fraction, increased LV wall thickness and diastolic dysfunction. Patients had reduced forced expiratory volume in 1 s (FEV1) and forced vital capacity (FVC) with normal FEV1/FVC, suggesting lung volume restriction. With this *de novo* series of CA patients, we confirm our previous findings (Monfort et al., 2020) that patients with CA display poor aerobic capacity. CA patients displayed reduced peak VO_2_ which was associated with impaired VO_2_-kinetics evaluated by mean response time (MRT) of VO_2_ increase and VO_2_ recovery delay. An hyperkinetic response of heart rate (peak heart rate/VO_2_ ratio) was observed in CA patients during exercise (Table 1). CA patients also displayed reduced O_2_ pulse at peak exercise, while neither O_2_ pulse decline during exercise nor paradoxical rebound during recovery were observed. Reduced predicted peak VO_2_ was associated with hyperventilation suggested by increased peak ventilatory equivalent ratio for O_2_ (V_E_/VO_2_) and CO_2_ (V_E_/VCO_2_), excess ventilation at anaerobic threshold (V_E_@ATVO_2_) and low end-tidal CO_2_ partial pressure P_ET_CO_2_.

**TABLE 1:** Main characteristics of cardiac ATTR amyloidosis patients according to ventilatory inefficiency V_E_-VCO_2_ slope.

	All ATTR patients (*n* = 41)	V_E_-VCO_2_ slope ≥ 36 (*n* = 27)	V_E_-VCO_2_ slope < 36 (*n* = 14)	*p*-value
Age, years	73 ± 7	73 ± 7	73 ± 7	0.977
Male gender, *n* (%)	36 (88)	23 (88)	13 (87)	0.613
BMI, kg/m^2^	24 ± 4	23 ± 3	25 ± 4	0.055
NYHA III/IV, *n* (%)	28 (68)	18 (69)	10 (67)	0.525
Baseline echocardiography				
IVS thickness, mm	15.4 ± 2.9	17.6 ± 3.0	15.4 ± 2.3	**0.029**
LV mass, g/m^2^	178 ± 53	194 ± 54	158 ± 47	0.058
LVEF, %	51 ± 16	50 ± 15	54 ± 16	0.431
Cardiac index, L min^−1^ m^2^	1.9 ± 0.4	1.8 ± 0.3	2.1 ± 0.5	0.077
E/e' ratio	14 ± 6	15 ± 5	13 ± 8	0.473
PASP, mmHg	38 ± 11	43 ± 12	34 ± 9	0.103
RAP, mmHg	8 ± 2	8 ± 3	6 ± 2	0.089
RV FAC, %	39 ± 12	37 ± 15	41 ± 9	0.626
RV S, cm s^−1^	10 ± 3	10 ± 3	10 ± 3	0.795
TAPSE	17 ± 6	15 ± 7	18 ± 6	0.349
Baseline lung function				
FEV_1_, % predicted	73 ± 15	68 ± 19	78 ± 15	0.075
FVC, % predicted	74 ± 14	71 ± 13	62 ± 12	0.088
FEV_1_/FVC, %	81 ± 8	82 ± 9	79 ± 7	0.216
CPET parameters				
Peak workload, %	50 ± 13	48 ± 12	55 ± 14	0.0.96
Peak VO_2_, ml kg^−1^ min^−1^	15.6 ± 3.4	15.0 ± 3.3	16.9 ± 3.1	0.063
Peak VO_2_, %	66 ± 15	62 ± 14	75 ± 10	**0.003**
VO_2_/W slope	10 ± 2	10 ± 3	11 ± 2	0.269
ATVO_2_, ml kg^−1^ min^−1^	11.3 ± 3.0	10.7 ± 3.0	12.4 ± 2.8	0.090
Peak RER	1.2 ± 0.1	1.2 ± 0.1	1.2 ± 0.1	0.733
Peak BF, min^−1^	36 ± 8	39 ± 8	34 ± 6	0.051
Peak tidal volume, L	1.4 ± 0.5	1.3 ± 0.4	1.6 ± 0.7	0.064
Excess V_E_@ATVO_2_, %	70 ± 24	77 ± 25	55 ± 15	**0.004**
P_ET_CO_2_@ATVO_2_, mmHg	31.4 ± 2.9	32.7 ± 4.4	32.8 ± 3.0	0.899
Peak P_ET_CO_2_, mmHg	32.4 ± 3.7	31.1 ± 3.4	34.8 ± 3.4	**0.002**
Ventilatory reserve, %	32 ± 18	32 ± 21	33 ± 12	0.722
EOV, *n* (%)	5 (12)	5 (33)	0 (0)	**0.004**
Peak V_E_/VO_2_	44 ± 7	46 ± 8	41 ± 5	**0.025**
Peak V_E_/VCO_2_	37 ± 5	39 ± 5	35 ± 3	**0.004**
V_E_ VCO_2_ slope	39 ± 5	42 ± 4	33 ± 2	**<0.0001**
Mean response time, sec	38 ± 11	36 ± 8	38 ± 8	0.497
Peak O_2_ pulse, %	75 ± 16	74 ± 16	76 ± 16	0.762
VO_2_ recovery delay, sec	51 ± 25	53 ± 27	42 ± 17	0.153
Resting heart rate, bpm	78 ± 16	75 ± 17	83 ± 16	0.150
Resting systolic AP, mm Hg	138 ± 24	139 ± 26	136 ± 22	0.709
Resting diastolic AP, mm Hg	85 ± 17	88 ± 18	80 ± 13	0.128
Peak heart rate, bpm	125 ± 20	120 ± 21	134 ± 15	**0.037**
Peak heart rate, %	85 ± 12	82 ± 12	91 ± 10	**0.020**
Peak heart rate/VO_2_ slope	8.7 ± 7.1	9.1 ± 8.8	8.2 ± 1.6	0.685
Peak systolic AP, mm Hg	176 ± 32	177 ± 34	173 ± 27	0.655
Peak diastolic AP, mm Hg	100 ± 19	103 ± 19	95 ± 18	0.179
Heart rate recovery 1 min; %	9 ± 5	8 ± 5	10 ± 5	0.411
Heart rate recovery 3 min; %	24 ± 10	20 ± 9	29 ± 7	**0.003**
Biological parameters				
eGFR, ml/min/1.72 m^2^	83 ± 41	84 ± 40	93 ± 45	0.565
Cardiac troponin T, ng/L	147 ± 155	158 ± 171	95 ± 103	0.273
NT-proBNP, ng/L	3,304 ± 2,274	3,917 ± 2,387	1833 ± 1971	0.068

Abbreviations: AP, arterial pressure; ATTR, transthyretin amyloidosis; BMI, body mass index; bpm, beat per minute; NYHA, New York Heart Association (NYHA) classification; IVS, interventricular septum thickness; LV, left ventricle; LVEF, left ventricular ejection fraction; E/e', early diastolic transmitral velocity to early mitral annulus diastolic velocity ratio; PASP, pulmonary artery systolic pressure, RAP, right atrial pressure; RV, FAC, right ventricular fractional area change; TAPSE, tricuspid annular plane systolic excursion; RV S, peak systolic tissue Doppler velocity of the tricuspid annulus; CPET, cardiopulmonary exercise testing; BF, breathing frequency; Vt, tidal volume; FEV_1_, forced expiratory volume in 1 s; FVC, forced vital capacity; VO_2_, oxygen uptake; RER, respiratory exchange ratio; V_E_, minute ventilation; VCO_2_, pulmonary carbon dioxide output; excess V_E_@ATVO_2_, excess ventilation at anaerobic threshold; P_ET_CO_2_, end-tidal CO_2_ partial pressure; EOV, exercise oscillatory ventilation; eGFR, estimated glomerular filtration rate; NT-proBNP, N-terminal pro B-type Natriuretic Peptide. Results are presented as mean ± standard deviation for quantitative variables, and as absolute value (percentage) for categorical variables; Bold highlights the significant *p*-value for statistical significance set at *p* < 0.05.

Compared with CA patients without ventilatory inefficiency (V_E_-VCO_2_ slope < 36), patients with ventilatory inefficiency (V_E_-VCO_2_ slope ≥ 36) had higher inter-ventricular septum thickness and left ventricular mass (Table 1). Pulmonary artery systolic pressure and right atrial pressure were higher in patients with ventilatory inefficiency, whereas no differences were found for parameters evaluating right ventricular function, such as right ventricular fractional area change (RV FAC), tricuspid annular plane systolic excursion (TAPSE); and systolic tissue Doppler velocity of the tricuspid annulus (S) (Table 1). Likewise, no differences were found for mean response time (MRT) of VO_2_ increase in patients with ventilatory inefficiency compared with CA patients without ventilatory inefficiency (Table 1). Compared with CA patients with V_E_-VCO_2_ slope < 36, CA patients with V_E_-VCO_2_ slope ≥ 36 displayed lower VO_2_ peak along with higher peak ventilatory equivalent ratio for O_2_ (V_E_/VO_2_ ratio) and prolonged heart rate recovery at 3 min post exercise (Table 1). Exercise oscillatory ventilation (EOV) was only observed in CA patients with ventilatory inefficiency (V_E_-VCO_2_ slope ≥ 36). Univariate and multivariate analysis of predictors of V_E_-VCO_2_ slope for the overall population is shown in Table 2. By multivariate analysis, only excess VE@ATVO_2_ (*β* = 0.127; *p* = 0.011) remained independent predictors of V_E_-VCO_2_ slope.

**TABLE 2:** Univariate and multivariate analysis of relationship between ventilatory inefficiency and main characteristics of cardiac ATTR amyloidosis patients (*n* = 41).

	Univariate analysis		Multivariate analysis	
	Pearson coefficient	p-value	*β* (95% CI)	p-value
PASP, mmHg	0.556	**0.011**		
RAP, mmHg	0.485	**0.030**		
FVC, % predicted	−0.331	**0.034**		
Peak workload, %	−0.352	**0.024**		
Peak VO_2_, ml kg^−1^ min^−1^	−0.376	**0.016**		
Predicted peak VO_2_, %	−0.359	**0.021**		
Peak BF, min^−1^	0.448	**0.003**		
Peak tidal volume, L	−0.395	**0.011**		
Excess V_E_@ATVO_2_, %	−0.441	**0.004**	**0.127 (0.034–0.220)**	**0.011**
Peak P_ET_CO_2_, mmHg	−0.462	**0.002**		
Peak V_E_/VCO_2_	0.380	**0.014**		
Peak heart rate, %	−0.324	**0.039**		
Heart rate recovery 3 min; %	−0.441	**0.004**		

Abbreviations: ATTR, transthyretin amyloidosis; BMI, body mass index; NYHA, New York Heart Association (NYHA) classification; IVS, interventricular septum thickness; LV, left ventricle; LVEF, left ventricular ejection fraction; E/e', early diastolic transmitral velocity to early mitral annulus diastolic velocity ratio; PASP, pulmonary artery systolic pressure, RAP, right atrial pressure; RV, FAC, right ventricular fractional area change; TAPSE, tricuspid annular plane systolic excursion; RV S, peak systolic tissue Doppler velocity of the tricuspid annulus; CPET, cardiopulmonary exercise testing; BF, breathing frequency; Vt, tidal volume; FEV_1_, forced expiratory volume in 1 s; FVC, forced vital capacity; VO_2_, oxygen uptake; RER, respiratory exchange ratio; V_E_, minute ventilation; VCO_2_, pulmonary carbon dioxide output; excess V_E_@ATVO_2_, excess ventilation at anaerobic threshold; P_ET_CO_2_, end-tidal CO_2_ partial pressure; eGFR, estimated glomerular filtration rate; NT-proBNP, N-terminal pro B-type Natriuretic Peptide. Variables with a *p*-value < 0.20 after univariate analysis were included in the multivariate model (forward). Bold highlights the significant *p*-value for statistical significance set at *p* < 0.05.

## Discussion

In our study, *p*. Val142Ile transthyretin CA displayed poor aerobic capacity, i.e., reduced predicted peak VO_2_, along with indirect evidence of hyperventilation such as increased peak ventilatory equivalent ratio for O_2_ (V_E_/VO_2_) and CO_2_ (V_E_/VCO_2_), excess ventilation at anaerobic threshold (V_E_@ATVO_2_) and low end-tidal CO_2_ partial pressure P_ET_CO_2_. VO2-kinetics was impaired in CA patients, suggesting limited performance of the cardiovascular system to rapidly alter oxygen supply to the working muscles as well as limited ability of the skeletal muscle to utilize oxygen. Compared with CA patients without ventilatory inefficiency, patients with ventilatory inefficiency had lower peak VO_2_ along with elevated ventilatory drive, exercise oscillatory ventilation (EOV) and prolonged post-exercise heart rate recovery. By multivariate analysis, only excess V_E_@AT_VO2_ remained an independent factor of ventilatory inefficiency.

Results of our study suggest that underperfusion of ventilated lung alveoli leading to V_A_/Q ratio mismatch may be involved in V_E_-VCO_2_ slope increase. This is supported by the findings that our CA patients displayed baseline right ventricular dysfunction along with abnormal O_2_ kinetics and reduced O_2_ pulse, which suggest limited blood flow increase to exercise. However, CA patients with V_E_-VCO_2_ slope ≥ 36 had similar baseline right ventricular dysfunction, O_2_ pulse and O_2_ kinetics impairment compared with CA patients with V_E_-VCO_2_ slope < 36. Of note, mean response time of VO_2_ increase at the start of exercise (MRT VO_2_) and VO_2_ recovery delay which has been closely related to exertional right ventricular dysfunction (Chatterjee et al., 2013) and poor cardiovascular response (Lauer, 2009) respectively, were not associated with ventilatory inefficiency in CA patients included in our study.

CA patients with ventilatory inefficiency had lower peak exercise end-tidal CO_2_ partial pressure (P_ET_CO_2_) compared with patients without ventilatory inefficiency. This finding may suggest pulmonary arterial pressure rise at peak exercise because the decrease of P_ET_CO_2_ from rest to peak exercise has been consistently associated with pulmonary hypertension. Overall, that right ventricular dysfunction and blunted cardiac output rise would participate to V_E_-VCO_2_ slope increase in *p*.Val142Ile transthyretin CA patients is not supported by our results.

According to the alveolar ventilation equation, V_E_-VCO_2_ slope is determined by two factors, which are the direction and magnitude of arterial CO_2_ partial pressure (PaCO_2_) changes and the fraction of tidal volume (the volume of air moved into or out of the lungs during a normal breath) that goes to dead space, i.e., the physiological dead space ratio (V_D_/V_T_ ratio) (Weatherald et al., 2018; Phillips et al., 2020). Hence, an excessive ventilatory drive leading to PaCO_2_ decrease would induce V_E_-VCO_2_ slope. In our patients, excessive ventilatory response to exercise was supported by increased ventilatory equivalent ratio for O_2_ (V_E_/VO_2_) and CO_2_ (V_E_/VCO_2_), low P_ET_CO_2_ levels and excess ventilation at the anaerobic threshold (V_E_@AT_VO2_). Excess V_E_@AT_VO2_ is a reliable marker of elevated ventilatory drive, which is not affected by transient hyperventilation early in exercise and by metabolic acidosis during high levels of exercise (Jones and Campbell, 1982; Fairshter et al., 1987; Péronnet et al., 2007; Péronnet and Aguilaniu, 2014). According to Jones and Campbell equation (Jones and Campbell, 1982), expected ventilation during exercise from baseline to anaerobic threshold may be calculated as a linear function of oxygen uptake (VO_2_). Minute-ventilation increase is called hyperpnea. Any minute-ventilation increase above the expected calculated value is referred as hyperventilation. In our study, compared with patients without inefficiency, CA patients with ventilatory inefficiency had higher excess V_E_@AT_VO2_, which was considered an independent factor of ventilatory inefficiency. Because arterial blood gas analysis was not performed in our study, whether excessive ventilatory stimulation in CA patients with ventilatory inefficiency would have driven down PaCO_2_, thus increasing V_E_-VCO_2_ slope remain speculative. Observation that CA patients with V_E_-VCO_2_ slope increase also displayed exercise oscillatory ventilation (EOV) may indicate major ventilatory instability, as both events share common determinants such as enhanced dead space, early occurrence of acidosis, and abnormal chemoreflex and/or metaboreflex activity.

Neurological manifestations including signs of neuropathy and dysautonomia due to amyloid damage of small myelinated and unmyelinated fibres are frequently reported in patients with transthyretin CA (Goldstein, 2016; Siddiqi and Ruberg, 2018; Gonzalez-Duarte et al., 2019; Kharoubi et al., 2021; Banydeen et al., 2022b). Small unmyelinated fibres amyloid damage may also alter mechano-reflex and metabo-reflex, so called ergoreflex, which modulates ventilation and cardiovascular function during exercise (Boyes et al., 2022). Ergoreflex sensitivity is typically overstimulated in heart failure contributing to sympathetic outflow increase and sympathovagal imbalance (Aimo et al., 2021; Boyes et al., 2022). Whereas ergoreflex sensitivity was not evaluated in our study in CA patients, we found that post-exercise heart rate recovery, a robust surrogate of cardiac autonomic imbalance, was markedly impaired in CA patients and correlated with ventilatory inefficiency. Hence, together with impaired autonomic cardiopulmonary regulation *via* peripheral (carotid body) and central (medullary) chemoreceptors, excessive stimulation of ergoreceptors (Aimo et al., 2021; Boyes et al., 2022) may have heightened the sympathetic activity converging to hyperventilation and increased V_E_-VCO_2_ slope. Further studies aimed to test whether the ergoreflex is impaired in CA patients are warranted.

Our study has several limitations. The sample size of our cohort is small that is mainly explained by the fact that transthyretin cardiac amyloidosis remains a rare disease even in expert centers evaluating cardiopulmonary function in these patients. Evaluation of right ventricular performance by echocardiography is challenging. While our results confirm that right ventricular performance is impaired in hereditary CA patients, no association with V_E_-VCO_2_ slope increase was observed, which contradicts previous findings. Invasive hemodynamic data of right ventricular and pulmonary hemodynamics should be implemented to better investigate the relationship between poor left and right-side heart function and ventilatory inefficiency. Impairment of VO_2_-kinetics was interpreted as being related to limited performance of the cardiovascular system to rapidly alter oxygen supply to the working muscles, but abnormal VO_2_-kinetics can also be related to a limited ability of the skeletal muscle to utilize oxygen.

Sympathetic outflow increase and sympathovagal imbalance was indirectly suggested by impaired post-exercise heart rate recovery. Direct measurement of sympathetic outflow, as well as ergoreflex and peripheral/central chemoreceptor sensitivity should be investigated in details in CA patients.

We have previously shown that blunted cardiac output rise in response to exercise participate to exercise intolerance in Afro-Caribbean patients with val122Ile (*p*.Val142Ile) ATTRv cardiomyopathy. It was shown that exercise-induced cardiac output rise in ATTRv patients was primarily achieved by increase of heart rate, while stroke volume (SV) failed to increase adequately relative to VO_2_ increase. In the present study, CA patients with higher V_E_-VCO_2_ slope demonstrated significantly lower peak heart rate in comparison with patients with relatively normal V_E_-VCO_2_ slope, which may suggest lower cardiac output at peak exercise. Hence, we cannot formally exclude that CA patients exhibit abnormal underperfusion of ventilated lung alveoli leading to V_D_/V_T_ ratio and V_E_-VCO_2_ slope increases.

Overall, the present study has added novel contribution to explain elevated V_E_-VCO_2_ slope in CA patients. Consistently with previous finding showing that CA patients can display abnormal pulmonary function (Banydeen et al., 2022a), our results suggest that excessive ventilatory drive could also participate to the elevated V_E_-VCO_2_ slope in CA patients.

## Conclusion

Results of our study confirm that patients with transthyretin CA show abnormal cardiopulmonary responses at CPET evaluation, which is characterized by reduced VO_2_ peak values V_E_-VCO_2_ slope increase. Our study provides new information regarding the mechanisms of ventilatory inefficiency in patients with transthyretin cardiac amyloidosis. Our data suggest that high ventilatory stimulation during exercise leading to hyperventilation is the main determinant of V_E_-VCO_2_ slope increase in hereditary transthyretin CA patients. The role of ergoreflex and peripheral/central chemoreceptor sensitivity, which are involved in excessive ventilatory response to exercise should be studied in details (Hearon et al., 2019).

## Data Availability

The raw data supporting the conclusion of this article will be made available by the authors, without undue reservation.

## Glossary

AT: anaerobic threshold
BF: breathing frequency
BMI: body mass index
bpm: beat per minute
BNP: brain natriuretic peptide
CA: cardiac amyloidosis
CI: chronotropic incompetence
CPET: cardiopulmonary exercise testing
E/e': early diastolic transmitral velocity to early mitral annulus diastolic velocity ratio
eGFR: estimated glomerular filtration rate
FEV1: forced expiratory volume in the first second
FVC: forced expiratory vital capacity
HR: heart rate
IVS: interventricular septum thickness
LVEF: left ventricular ejection fraction
MRT: mean response time
MVV: maximal voluntary ventilation
NYHA: New York heart association
PaCO_2_: arterial CO_2_ partial pressure
PASP: pulmonary artery systolic pressure
P_ET_CO_2_: end-tidal CO_2_ partial pressure
RAP: Right atrial pressure
RV FAC: right ventricular fractional area change
RV S: peak systolic tissue Doppler velocity of the tricuspid annulus
RER: respiratory exchange ratio
SPECT: single-photon emission computed tomography
SV: stroke volume
SVR: systemic vascular resistance
TAPSE: tricuspid annular plane systolic excursion
V_A_/Q: pulmonary ventilation-to-perfusion
VCO_2_: carbon dioxide output
V_D_: dead volume
V_T_: tidal volume
V_D_/V_T_: physiological dead space
VE: minute ventilation
VO_2_: oxygen uptake
